# Supplementary material for: The gut microbiome and early-life growth in a population with high prevalence of stunting
Source: Nat Commun. 2023 Feb 14;14:654. doi: 10.1038/s41467-023-36135-6 (PMC9929340; doi:10.1038/s41467-023-36135-6)
Supplement: Supplementary file 1 — Supplementary Information [file 41467_2023_36135_MOESM1_ESM.pdf]

Supplementary Information

The gut microbiome and early-life growth in a population with high prevalence of stunting

Robertson et al.

Supplementary Table 1. Sample breakdown in each age category

	Maternal HIV status	
	**Positive**	**Negative**
**1 month**	36	107
**2 months**	29	48
**3 months**	70	117
**6 months**	63	55
**12 months**	100	27
**18 months**	93	130

Supplementary Table 2. Baseline characteristics of infants in SHINE trial and microbiome sub-study

	SHINE	Microbiome sub-study
	N=4727	N=335
**Mothers HIV+**	738 (15.6%)	99 (29.6%)
**Female**	2329 (49.5%)	147 (43.9%)
**Birthweight, kg (sd)**	3.07 (0.5)	3.09 (0.49)
**Low birthweight**	410 (9.7%)	27 (8.2%)
**Gestational age, weeks (sd)**	38.4 (4.03)	39.2 (3.43)
**Standard vaginal delivery**	4000 (92.5%)	308 (93.9%)
**Institutional delivery**	3752 (88.2%)	294 (89.9%)
**Exclusive breastfeeding**	2397 (87.9%)	292 (91.0%)
**LAZ - 1 month, (sd)**	-0.9 (1.35)	-0.96 (1.25)
**LAZ - 3 months, (sd)**	-0.91 (1.33)	-0.94 (1.18)
**LAZ - 6 months, (sd)**	-0.95 (1.25)	-0.91 (1.15)
**LAZ - 12 months, (sd)**	-1.28 (1.22)	-1.21 (1.05)
**LAZ - 18 months, (sd)**	-1.58 (1.13)	-1.48 (1.07)
**Trial arm**		
SOC	1126 (23.8%)	88 (26.3%)
IYCF	1121 (23.7%)	93 (27.8%)
WASH	1201 (25.4%)	71 (21.2%)
WASH+IYCF	1279 (27.1%)	83 (24.8%)
**Household size, [IQR]**	5.00 [3.00;6.00]	5.00 [4.00;6.00]
**Mother age, years (sd)**	26.2 (6.66)	28.3 (6.35)
**Mother height, cm (sd)**	160.1 (5.94)	161 (5.78)
**Parity, [IQR]**	2.00 [1.00;3.00]	2.00 [1.00;3.00]
**Maternal MUAC, cm (sd)**	26.4 (3.08)	27.1 (3.23)
**Maternal education, years (sd)**	9.52 (1.85)	9.39 (1.90)
**Mothers employed**	383 (8.7%)	30 (9.17%)
**Open defacation in household**	1952 (44.8%)	154 (47.1%)
**Any latrine in household**	1743 (39.8%)	128 (40.3%)
**Improved latrine in household**	1535 (35.1%)	119 (37.4%)
**Improved floor in household**	2370 (54.2%)	183 (56.5%)
**Household owns chickens**	3512 (79.0%)	264 (80.5%)
**Livestock in household**	1691 (36.6%)	146 (44.0%)

Supplementary Table 3. Detected Eukaryota and Archaea species prior to prevalence filtering

**Eukaryota**	**Archaea**
Blastocystis sp subtype 1	*Methanobrevibacter oralis*
Candida albicans	*Methanobrevibacter smithii*
Cryptosporidium hominis	*Methanobrevibacter woesei*
Cryptosporidium meleagridis	*Methanosphaera stadtmanae*
Cryptosporidium parvum	
Giardia intestinalis	
Saccharomyces cerevisiae	

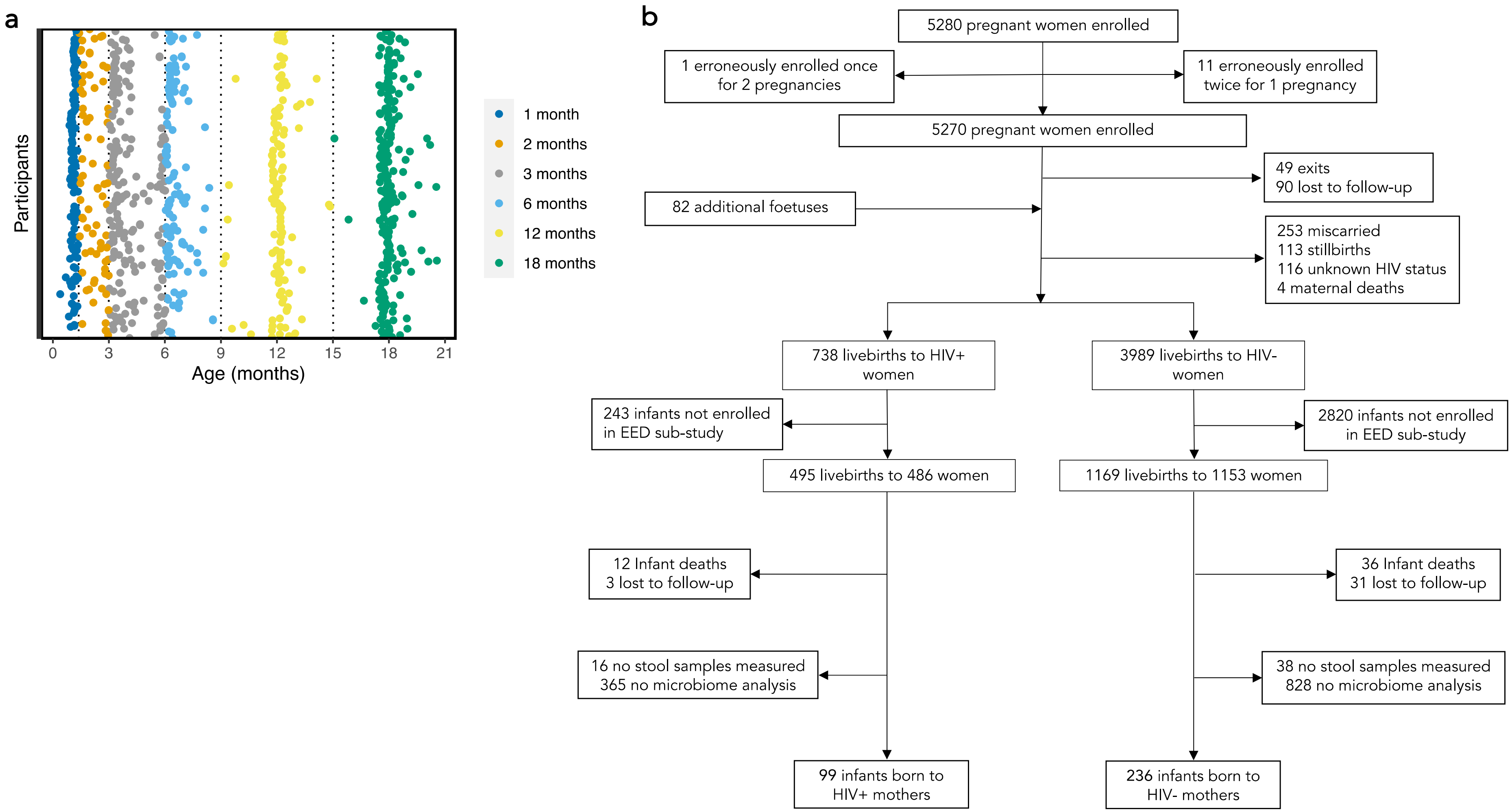
a
Participants
Age (months)
0
3
6
9
12
15
18
21
1 month
2 months
3 months
6 months
12 months
18 months
b
5280 pregnant women enrolled
1 erroneously enrolled once for 2 pregnancies
11 erroneously enrolled twice for 1 pregnancy
5270 pregnant women enrolled
49 exits
90 lost to follow-up
82 additional foetuses
253 miscarried
113 stillbirths
116 unknown HIV status
4 maternal deaths
738 livebirths to HIV+ women
3989 livebirths to HIV- women
243 infants not enrolled in EED sub-study
2820 infants not enrolled in EED sub-study
495 livebirths to 486 women
1169 livebirths to 1153 women
12 Infant deaths
3 lost to follow-up
36 Infant deaths
31 lost to follow-up
16 no stool samples measured
365 no microbiome analysis
38 no stool samples measured
828 no microbiome analysis
99 infants born to HIV+ mothers
236 infants born to HIV- mothers

Supplementary Figure 1. Participants and samples included in the study. 875 stool samples collected from 335 unique infants underwent whole metagenome shotgun sequencing and were categorized into 6 age groups (a). CONSORT diagram of the participants from the SHINE trial included in the infant microbiome sub-study (b).

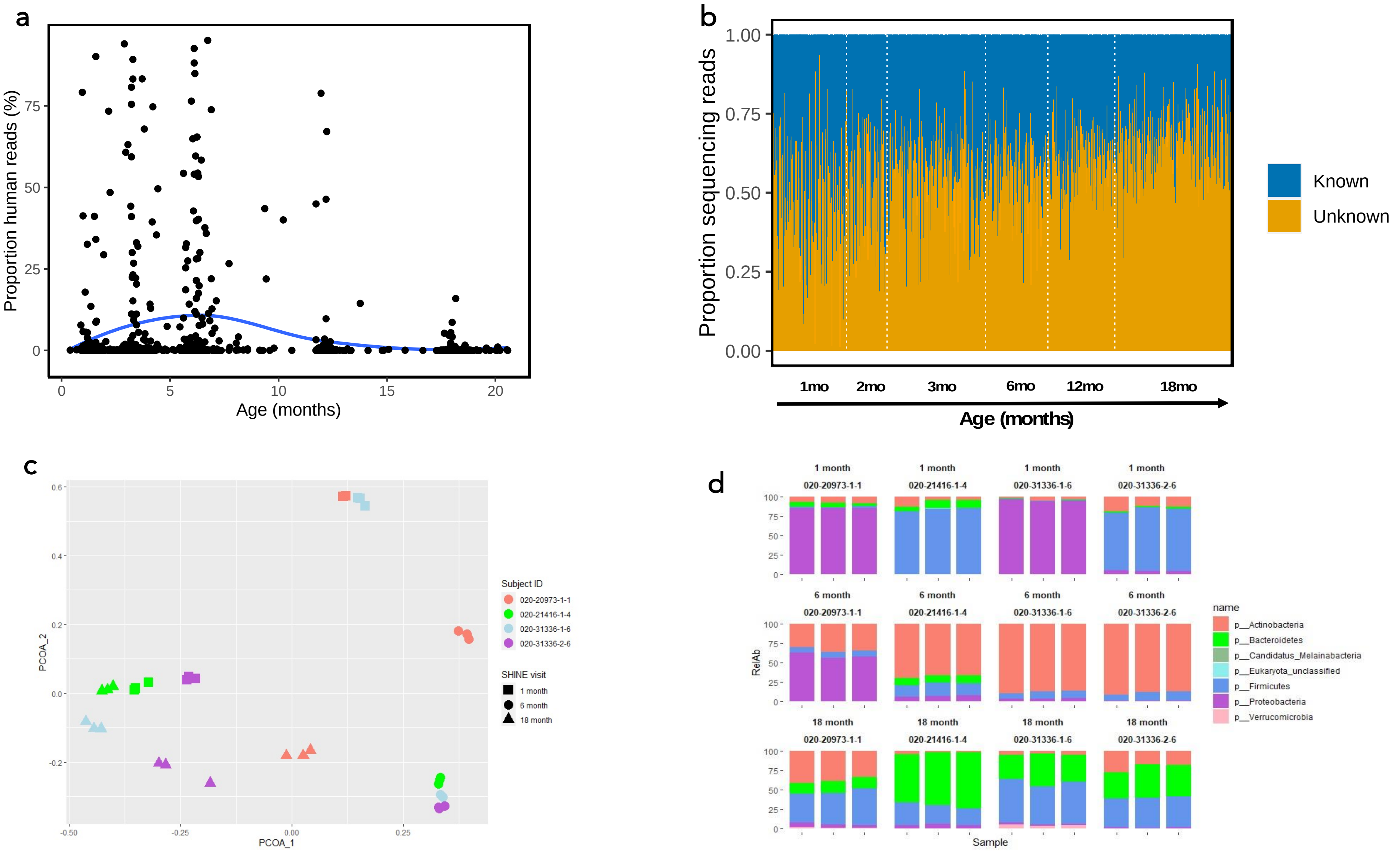
a
Proportion human reads (%)
Age (months)
0
5
10
15
20
25
50
75
b
Proportion sequencing reads
0.00
0.25
0.50
0.75
1.00
Known
Unknown
1mo
2mo
3mo
6mo
12mo
18mo
Age (months)
c
PCOA_2
PCOA_1
-0.50
-0.25
0.00
0.25
-0.2
0.0
0.2
0.4
0.6
Subject ID
020-20973-1-1
020-21416-1-4
020-31336-1-6
020-31336-2-6
SHINE visit
1 month
6 month
18 month
d
1 month
6 month
18 month
020-20973-1-1
020-21416-1-4
020-31336-1-6
020-31336-2-6
RelAb
Sample
name
p__Actinobacteria
p__Bacteroidetes
p__Candidatus_Melainabacteria
p__Eukaryota_unclassified
p__Firmicutes
p__Proteobacteria
p__Verrucomicrobia

Supplementary Figure 2. Whole metagenome sequencing performance. A median of 0.05% sequencing reads were assigned to the human genome in each sample (a), which varied by age at stool sample collection (95% confidence interval in grey shaded area). The percentage of sequencing reads that could be aligned to known sequences using the MetaPhlAn3 and HUMAnN3 pipelines decreased in stool samples collected at older ages (b). PCoA (c) and phylum relative abundances (d) of sequencing sample replicates showed high reproducibility and little technical variation.

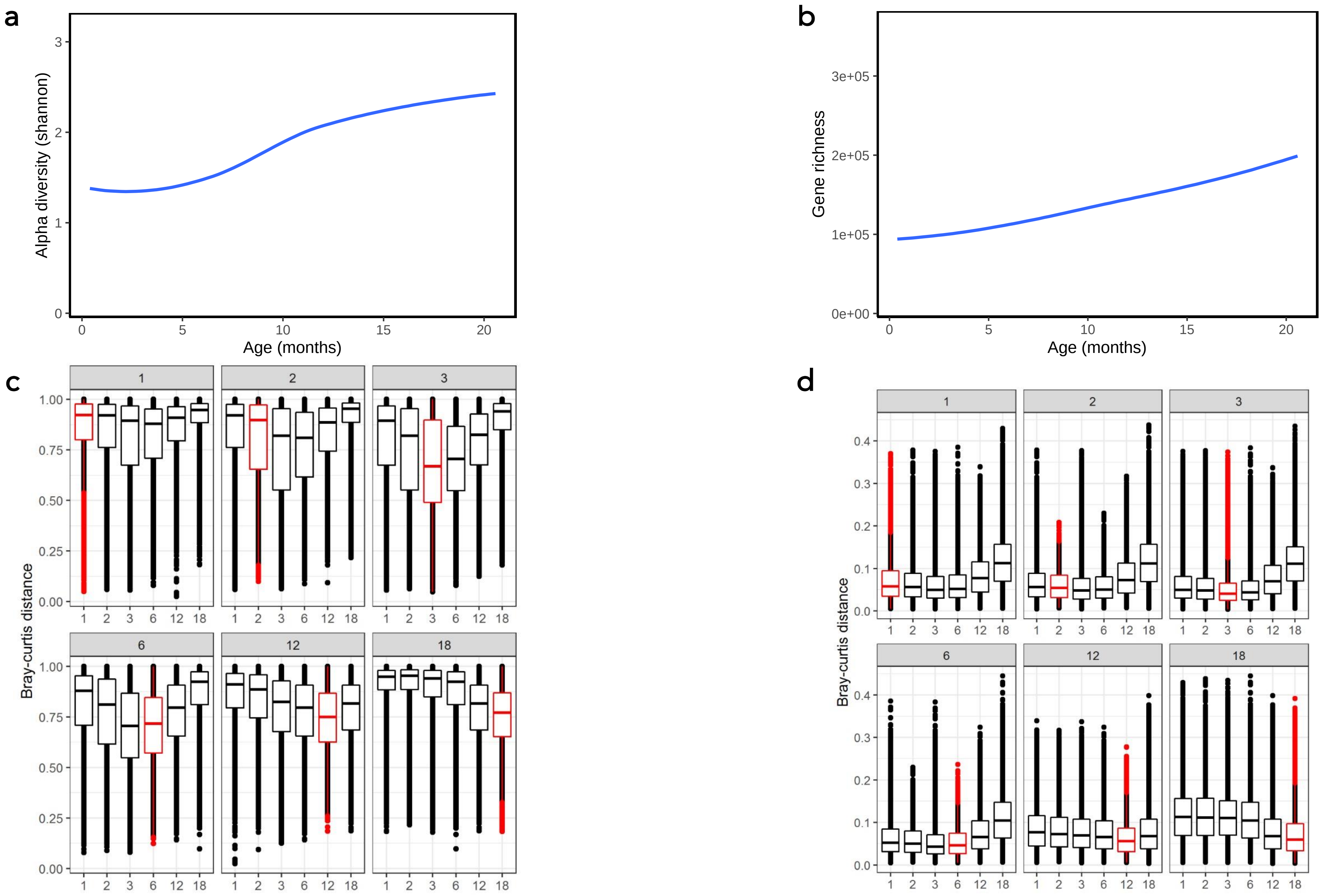

a
Alpha diversity (shannon)
Age (months)
b
Gene richness
3e+05
2e+05
1e+05
0e+00
Age (months)
c
Bray-curtis distance
1.00
0.75
0.50
0.25
0.00
1 2 3 6 12 18
d
Bray-curtis distance
0.4
0.3
0.2
0.1
0.0
1 2 3 6 12 18

Supplementary Figure 3. Diversity metrics in entire dataset. Shannon alpha diversity (a) and gene richness (b) across the entire dataset revealed stable diversity up to 4-5 months of age followed by rapid taxonomic and functional diversification (Lines represent smoothed conditional means and grey shaded areas represent 95% confidence intervals). Bray-curtis distances between samples at within and across each age visit showed low inter-individual variability in species composition which increase with age (c), and high inter-individual variation in metagenome pathways (d; the band indicates the median, the box indicates the first and third quartiles and the whiskers indicate ± 1.5 × interquartile range. Red boxes indicate comparisons of distance within age-groups). n=875 samples

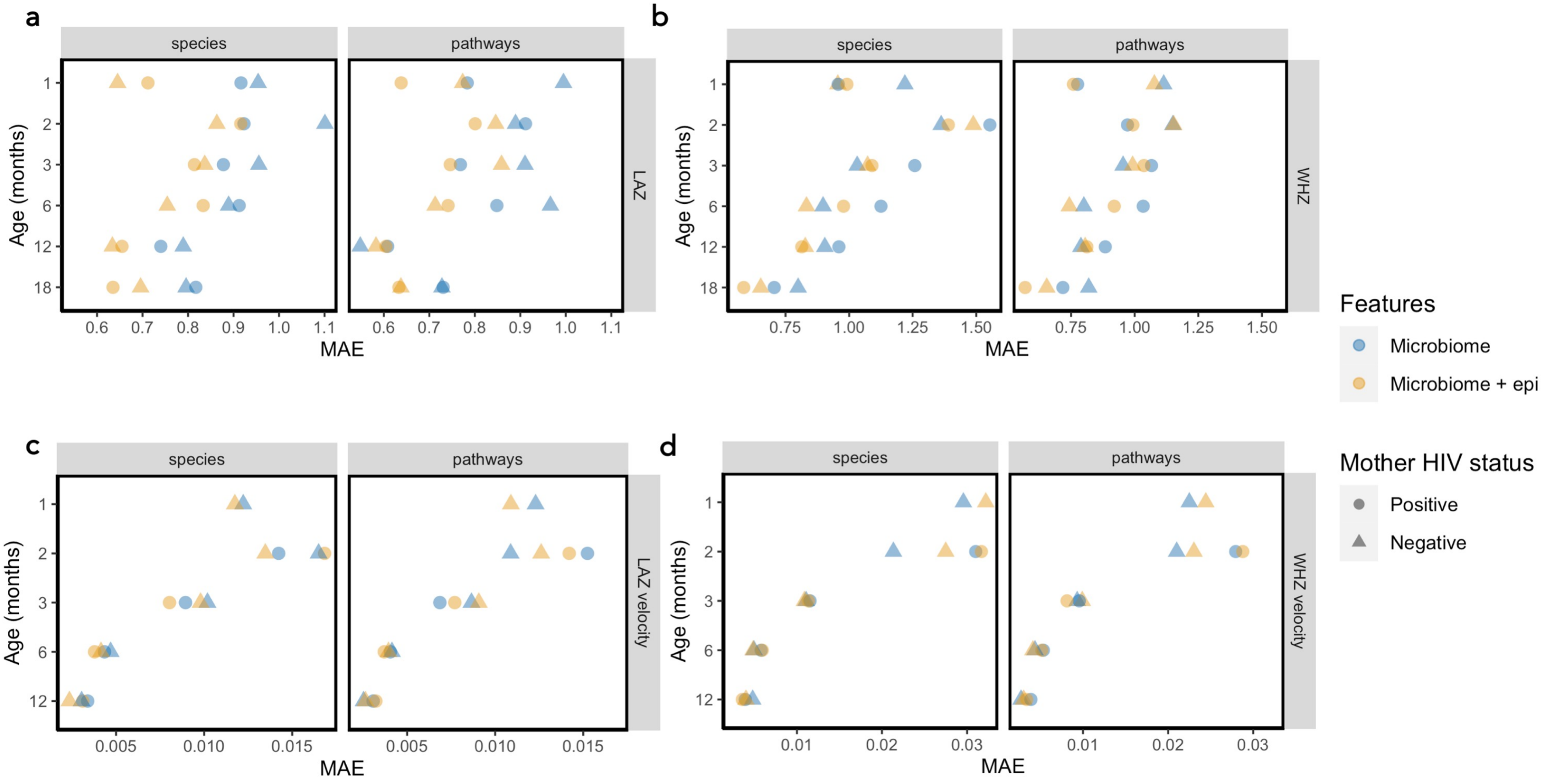
a
species
pathways
LAZ
Age (months)
1
2
3
6
12
18
0.6 0.7 0.8 0.9 1.0 1.1
MAE
b
species
pathways
WHZ
0.75 1.00 1.25 1.50
c
species
pathways
LAZ velocity
0.005 0.010 0.015
d
species
pathways
WHZ velocity
0.01 0.02 0.03
Features
Microbiome
Microbiome + epi
Mother HIV status
Positive
Negative

Supplementary Figure 4. XGBoost model performance metrics as assessed by mean absolute error. Mean absolute error (MAE) in XGBoost model performances in models predicting LAZ (a), WHZ (b), LAZ velocity (c) and WHZ velocity (d) stratified by maternal HIV status and age categories. Models were run using microbiome features alone (species or metagenomic pathways) and in combination with epidemiological variables (epi). Growth velocity was defined as LAZ/WHZ units of change per day between the specified study visit and the subsequent study visit.

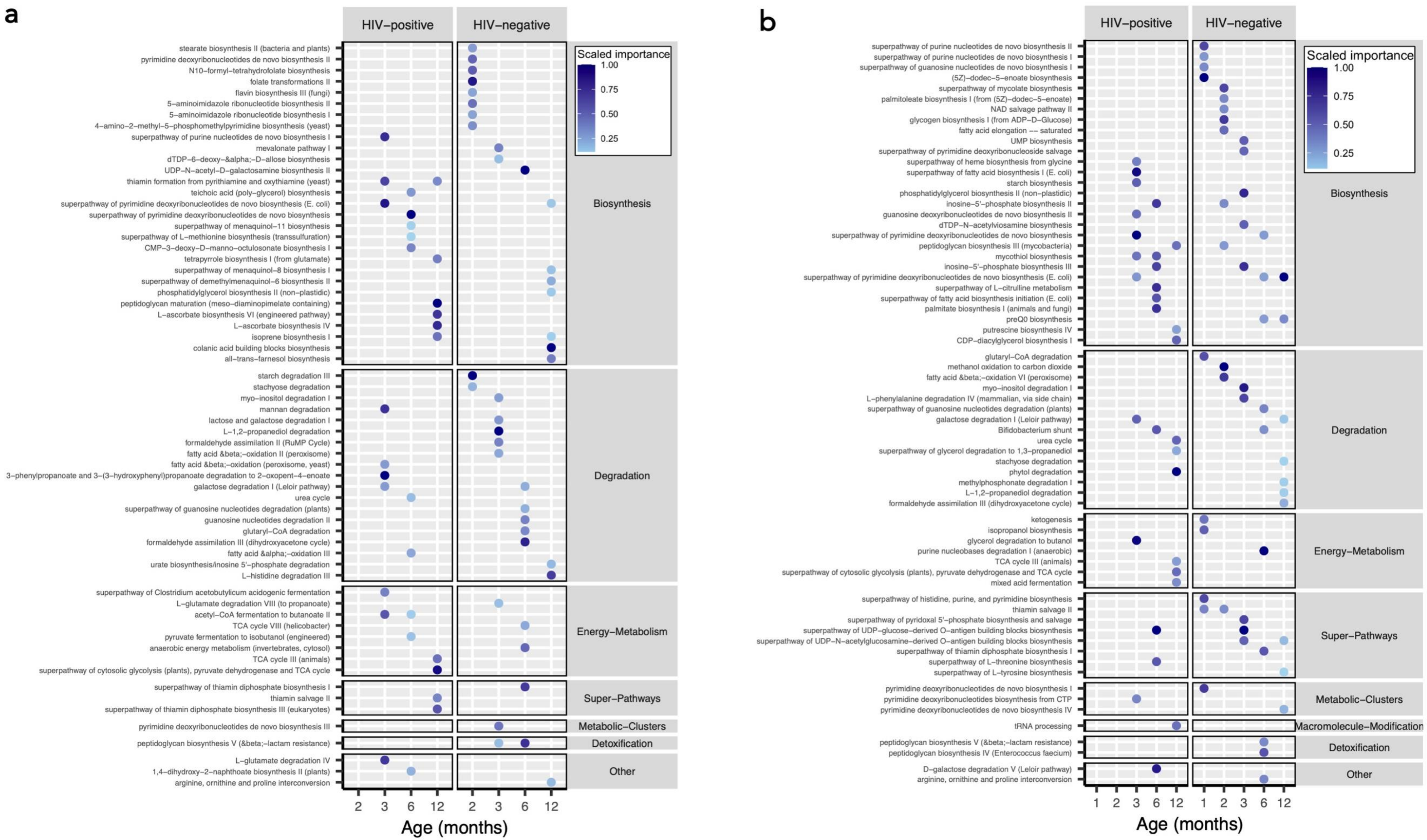
a
HIV−positive
HIV−negative
Scaled importance
1.00
0.75
0.50
0.25
stearate biosynthesis II (bacteria and plants)
pyrimidine deoxyribonucleotides de novo biosynthesis II
N10−formyl−tetrahydrofolate biosynthesis
folate transformations II
flavin biosynthesis III (fungi)
5−aminoimidazole ribonucleotide biosynthesis II
5−aminoimidazole ribonucleotide biosynthesis I
4−amino−2−methyl−5−phosphomethylpyrimidine biosynthesis (yeast)
superpathway of purine nucleotides de novo biosynthesis I
mevalonate pathway I
dTDP−6−deoxy−α−D−allose biosynthesis
UDP−N−acetyl−D−galactosamine biosynthesis II
thiamin formation from pyrithiamine and oxythiamine (yeast)
teichoic acid (poly−glycerol) biosynthesis
superpathway of pyrimidine deoxyribonucleotides de novo biosynthesis (E. coli)
superpathway of pyrimidine deoxyribonucleotides de novo biosynthesis
superpathway of menaquinol−11 biosynthesis
superpathway of L−methionine biosynthesis (transsulfuration)
CMP−3−deoxy−D−manno−octulosonate biosynthesis I
tetrapyrrole biosynthesis I (from glutamate)
superpathway of menaquinol−8 biosynthesis I
superpathway of demethylmenaquinol−6 biosynthesis II
phosphatidylglycerol biosynthesis II (non−plastidic)
peptidoglycan maturation (meso−diaminopimelate containing)
L−ascorbate biosynthesis VI (engineered pathway)
L−ascorbate biosynthesis IV
isoprene biosynthesis I
colanic acid building blocks biosynthesis
all−trans−farnesol biosynthesis
Biosynthesis
starch degradation III
stachyose degradation
myo−inositol degradation I
mannan degradation
lactose and galactose degradation I
L−1,2−propanediol degradation
formaldehyde assimilation II (RuMP Cycle)
fatty acid β−oxidation II (peroxisome)
fatty acid β−oxidation (peroxisome, yeast)
3−phenylpropanoate and 3−(3−hydroxyphenyl)propanoate degradation to 2−oxopent−4−enoate
galactose degradation I (Leloir pathway)
urea cycle
superpathway of guanosine nucleotides degradation (plants)
guanosine nucleotides degradation II
glutaryl−CoA degradation
formaldehyde assimilation III (dihydroxyacetone cycle)
fatty acid α−oxidation III
urate biosynthesis/inosine 5'−phosphate degradation
L−histidine degradation III
Degradation
superpathway of Clostridium acetobutylicum acidogenic fermentation
L−glutamate degradation VIII (to propanoate)
acetyl−CoA fermentation to butanoate II
TCA cycle VIII (helicobacter)
pyruvate fermentation to isobutanol (engineered)
anaerobic energy metabolism (invertebrates, cytosol)
TCA cycle III (animals)
superpathway of cytosolic glycolysis (plants), pyruvate dehydrogenase and TCA cycle
Energy−Metabolism
superpathway of thiamin diphosphate biosynthesis I
thiamin salvage II
superpathway of thiamin diphosphate biosynthesis III (eukaryotes)
Super−Pathways
pyrimidine deoxyribonucleotides de novo biosynthesis III
Metabolic−Clusters
peptidoglycan biosynthesis V (β−lactam resistance)
Detoxification
L−glutamate degradation IV
1,4−dihydroxy−2−naphthoate biosynthesis II (plants)
arginine, ornithine and proline interconversion
Other
2 3 6 12 2 3 6 12
Age (months)
b
HIV−positive
HIV−negative
Scaled importance
1.00
0.75
0.50
0.25
superpathway of purine nucleotides de novo biosynthesis II
superpathway of purine nucleotides de novo biosynthesis I
superpathway of guanosine nucleotides de novo biosynthesis I
(5Z)−dodec−5−enoate biosynthesis
superpathway of mycolate biosynthesis
palmitoleate biosynthesis I (from (5Z)−dodec−5−enoate)
NAD salvage pathway II
glycogen biosynthesis I (from ADP−D−Glucose)
fatty acid elongation −− saturated
UMP biosynthesis
superpathway of pyrimidine deoxyribonucleoside salvage
superpathway of heme biosynthesis from glycine
superpathway of fatty acid biosynthesis I (E. coli)
starch biosynthesis
phosphatidylglycerol biosynthesis II (non−plastidic)
inosine−5'−phosphate biosynthesis II
guanosine deoxyribonucleotides de novo biosynthesis II
dTDP−N−acetylviosamine biosynthesis
superpathway of pyrimidine deoxyribonucleotides de novo biosynthesis
peptidoglycan biosynthesis III (mycobacteria)
mycothiol biosynthesis
inosine−5'−phosphate biosynthesis III
superpathway of pyrimidine deoxyribonucleotides de novo biosynthesis (E. coli)
superpathway of L−citrulline metabolism
superpathway of fatty acid biosynthesis initiation (E. coli)
palmitate biosynthesis I (animals and fungi)
preQ0 biosynthesis
putrescine biosynthesis IV
CDP−diacylglycerol biosynthesis I
Biosynthesis
glutaryl−CoA degradation
methanol oxidation to carbon dioxide
fatty acid β−oxidation VI (peroxisome)
myo−inositol degradation I
L−phenylalanine degradation IV (mammalian, via side chain)
superpathway of guanosine nucleotides degradation (plants)
galactose degradation I (Leloir pathway)
Bifidobacterium shunt
urea cycle
superpathway of glycerol degradation to 1,3−propanediol
stachyose degradation
phytol degradation
methylphosphonate degradation I
L−1,2−propanediol degradation
formaldehyde assimilation III (dihydroxyacetone cycle)
Degradation
ketogenesis
isopropanol biosynthesis
glycerol degradation to butanol
purine nucleobases degradation I (anaerobic)
TCA cycle III (animals)
superpathway of cytosolic glycolysis (plants), pyruvate dehydrogenase and TCA cycle
mixed acid fermentation
Energy−Metabolism
superpathway of histidine, purine, and pyrimidine biosynthesis
thiamin salvage II
superpathway of pyridoxal 5'−phosphate biosynthesis and salvage
superpathway of UDP−glucose−derived O−antigen building blocks biosynthesis
superpathway of UDP−N−acetylglucosamine−derived O−antigen building blocks biosynthesis
superpathway of thiamin diphosphate biosynthesis I
superpathway of L−threonine biosynthesis
superpathway of L−tyrosine biosynthesis
Super−Pathways
pyrimidine deoxyribonucleotides de novo biosynthesis I
pyrimidine deoxyribonucleotides biosynthesis from CTP
pyrimidine deoxyribonucleotides de novo biosynthesis IV
Metabolic−Clusters
tRNA processing
Macromolecule−Modification
peptidoglycan biosynthesis V (β−lactam resistance)
peptidoglycan biosynthesis IV (Enterococcus faecium)
Detoxification
D−galactose degradation V (Leloir pathway)
arginine, ornithine and proline interconversion
Other
1 2 3 6 12 1 2 3 6 12
Age (months)

Supplementary Figure 5. Top ranked pathways in XGBoost models predicting growth velocity. Top ranked features in XGBoost model predictions of LAZ velocity (a) and WHZ velocity (b) stratified by maternal HIV status. Only features from XGBoost models with pseudo-R^2 > 0 are plotted.

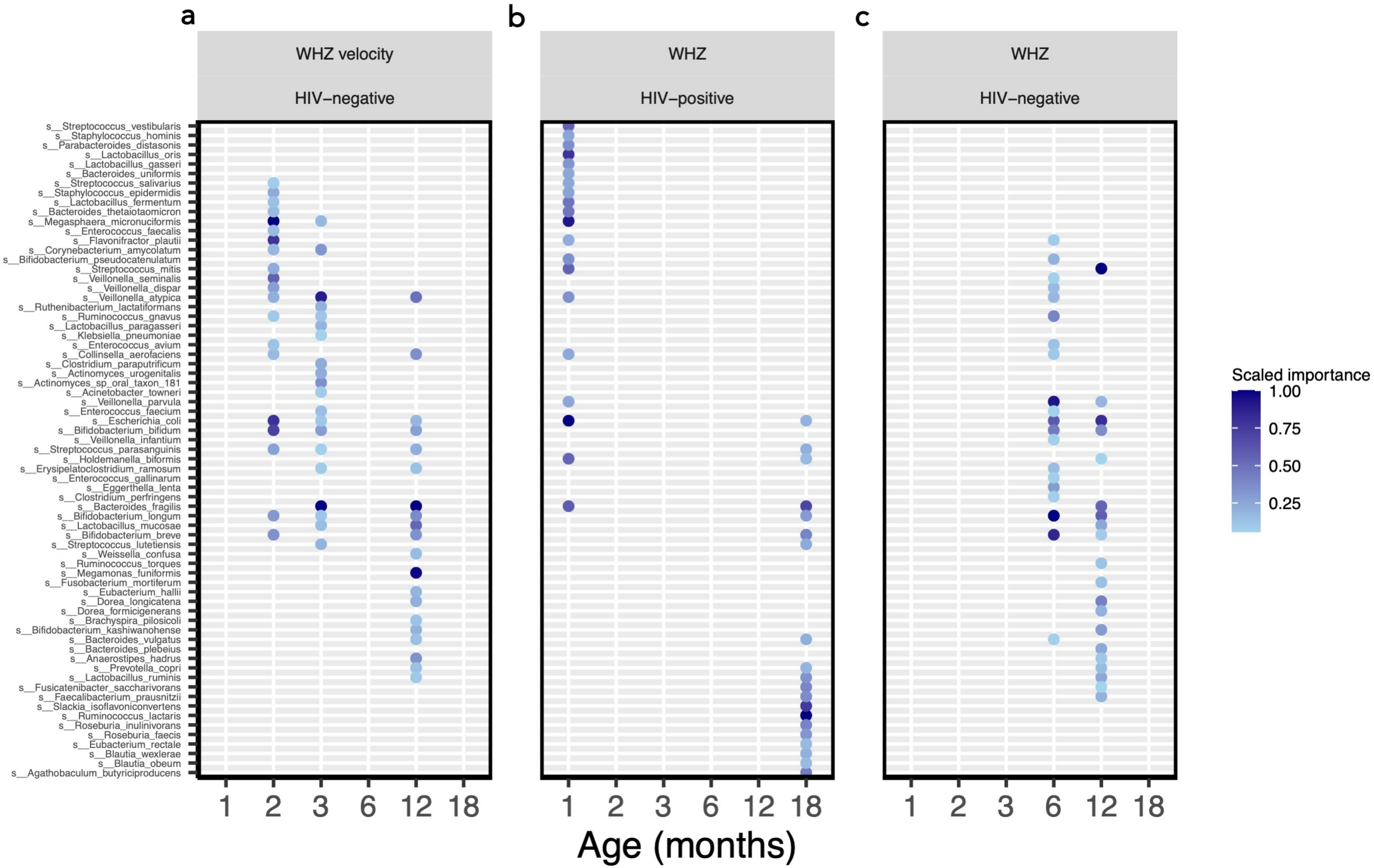
a
b
c
WHZ velocity
HIV−negative
WHZ
HIV−positive
WHZ
HIV−negative
s__Streptococcus_vestibularis
s__Staphylococcus_hominis
s__Parabacteroides_distasonis
s__Lactobacillus_oris
s__Lactobacillus_gasseri
s__Bacteroides_uniformis
s__Streptococcus_salivarius
s__Staphylococcus_epidermidis
s__Lactobacillus_fermentum
s__Bacteroides_thetaiotaomicron
s__Megasphaera_micronuciformis
s__Enterococcus_faecalis
s__Flavonifractor_plautii
s__Corynebacterium_amycolatum
s__Bifidobacterium_pseudocatenulatum
s__Streptococcus_mitis
s__Veillonella_seminalis
s__Veillonella_dispar
s__Veillonella_atypica
s__Ruthenibacterium_lactatiformans
s__Ruminococcus_gnavus
s__Lactobacillus_paragasseri
s__Klebsiella_pneumoniae
s__Enterococcus_avium
s__Collinsella_aerofaciens
s__Clostridium_paraputrificum
s__Actinomyces_urogenitalis
s__Actinomyces_sp_oral_taxon_181
s__Acinetobacter_towneri
s__Veillonella_parvula
s__Enterococcus_faecium
s__Escherichia_coli
s__Bifidobacterium_bifidum
s__Veillonella_infantium
s__Streptococcus_parasanguinis
s__Holdemanella_biformis
s__Erysipelatoclostridium_ramosum
s__Enterococcus_gallinarum
s__Eggerthella_lenta
s__Clostridium_perfringens
s__Bacteroides_fragilis
s__Bifidobacterium_longum
s__Lactobacillus_mucosae
s__Bifidobacterium_breve
s__Streptococcus_lutetiensis
s__Weissella_confusa
s__Ruminococcus_torques
s__Megamonas_funiformis
s__Fusobacterium_mortiferum
s__Eubacterium_hallii
s__Dorea_longicatena
s__Dorea_formicigenerans
s__Brachyspira_pilosicoli
s__Bifidobacterium_kashiwanohense
s__Bacteroides_vulgatus
s__Bacteroides_plebeius
s__Anaerostipes_hadrus
s__Prevotella_copri
s__Lactobacillus_ruminis
s__Fusicatenibacter_saccharivorans
s__Faecalibacterium_prausnitzii
s__Slackia_isoflavoniconvertens
s__Ruminococcus_lactaris
s__Roseburia_inulinivorans
s__Roseburia_faecis
s__Eubacterium_rectale
s__Blautia_wexlerae
s__Blautia_obeum
s__Agathobaculum_butyriciproducens
1 2 3 6 12 18
1 2 3 6 12 18
1 2 3 6 12 18
Age (months)
Scaled importance
1.00
0.75
0.50
0.25

Supplementary Figure 6. Top ranked species in XGBoost models predicting growth. Top ranked features in XGBoost model predictions of WHZ velocity (a) and WHZ in children born to HIV+ (b) and HIV- mothers (c). Only features from XGBoost models with pseudo-R^2 > 0 are plotted.
