# Supplementary material for: The gut microbiome and early-life growth in a population with high prevalence of stunting
Source: Nat Commun. 2023 Feb 14;14:654. doi: 10.1038/s41467-023-36135-6 (PMC9929340; doi:10.1038/s41467-023-36135-6)
Supplement: Supplementary file 3 — Description of Additional Supplementary Files [file 41467_2023_36135_MOESM3_ESM.pdf]

Description of Supplementary Data Files

The gut microbiome and early-life growth in a population with high prevalence of stunting

Robertson et al.

Supplementary Data File 1. Multivariate regression analysis examining the effect of maternal HIV infection, age (days), exclusive breastfeeding status, delivery mode and trial arm on taxonomic microbiome composition (multivariate linear regression analyses adjusted for multiple comparisons using Benjamini–Hochberg correction; two-sided p-values and q-values)

Supplementary Data File 2. Multivariate regression analysis examining the effect of maternal HIV infection, age (days), exclusive breastfeeding status, delivery mode and trial arm on gene pathway microbiome composition (multivariate linear regression analyses adjusted for multiple comparisons using Benjamini–Hochberg correction: two-sided p-values and q-values)

Supplementary Data File 3. Epidemiological variables included in XGBoost models
