# Supplementary material for: The gut microbiome and early-life growth in a population with high prevalence of stunting
Source: Nat Commun. 2023 Feb 14;14:654. doi: 10.1038/s41467-023-36135-6 (PMC9929340; doi:10.1038/s41467-023-36135-6)
Supplement: Supplementary file 7 — Reporting Summary [file 41467_2023_36135_MOESM7_ESM.pdf]

nature portfolio

Corresponding author(s): Prof. Amee Manges

Last updated by author(s): Dec 20, 2022

Reporting Summary

Nature Portfolio wishes to improve the reproducibility of the work that we publish. This form provides structure for consistency and transparency in reporting. For further information on Nature Portfolio policies, see our Editorial Policies and the Editorial Policy Checklist.

Statistics

For all statistical analyses, confirm that the following items are present in the figure legend, table legend, main text, or Methods section.

n/a	Confirmed	
☐	☒	The exact sample size (*n*) for each experimental group/condition, given as a discrete number and unit of measurement
☐	☒	A statement on whether measurements were taken from distinct samples or whether the same sample was measured repeatedly
☐	☒	The statistical test(s) used AND whether they are one- or two-sided<br>*Only common tests should be described solely by name; describe more complex techniques in the Methods section.*
☐	☒	A description of all covariates tested
☐	☒	A description of any assumptions or corrections, such as tests of normality and adjustment for multiple comparisons
☐	☒	A full description of the statistical parameters including central tendency (e.g. means) or other basic estimates (e.g. regression coefficient) AND variation (e.g. standard deviation) or associated estimates of uncertainty (e.g. confidence intervals)
☐	☒	For null hypothesis testing, the test statistic (e.g. *F*, *t*, *r*) with confidence intervals, effect sizes, degrees of freedom and *P* value noted<br>*Give P values as exact values whenever suitable.*
☐	☒	For Bayesian analysis, information on the choice of priors and Markov chain Monte Carlo settings
☐	☒	For hierarchical and complex designs, identification of the appropriate level for tests and full reporting of outcomes
☐	☒	Estimates of effect sizes (e.g. Cohen's *d*, Pearson's *r*), indicating how they were calculated

Our web collection on statistics for biologists contains articles on many of the points above.

Software and code

Policy information about availability of computer code

Data collection	No code used for data collection. Questionnaire data were collected on written-forms and doubly-entered onto an SQL database.
Data analysis	The code for the implementation of xgboost and the ALE plots is available at https://github.com/ThadEdens/shine-analysis.

For manuscripts utilizing custom algorithms or software that are central to the research but not yet described in published literature, software must be made available to editors and reviewers. We strongly encourage code deposition in a community repository (e.g. GitHub). See the Nature Portfolio guidelines for submitting code & software for further information.

Data

Policy information about availability of data

All manuscripts must include a data availability statement. This statement should provide the following information, where applicable:

- Accession codes, unique identifiers, or web links for publicly available datasets
- A description of any restrictions on data availability
- For clinical datasets or third party data, please ensure that the statement adheres to our policy

The raw metagenome sequencing data generated in this study have been deposited in the European Bioinformatics Database under accession code PRJEB51728 (https://www.ebi.ac.uk/ena/browser/view/PRJEB51728). The UniRef90 database used to annotate metabolic pathways is publicly available (https://www.uniprot.org/help/uniref). Epidemiologic data files and final processed and annotated metagenome sequencing data files (taxa and pathways) are available at 10.5281/zenodo.7471082 (https://zenodo.org/record/7471082#.Y6RrNH3MI2w).

Human research participants

Policy information about studies involving human research participants and Sex and Gender in Research.

Reporting on sex and gender	All data in this study is generated from children from 1-18 months of age. The infant sex breakdown is reported. No data has previously reported large-scale differences in the the developing microbiomes of male vs female infants and therefore sex differences were not directly compared.
Population characteristics	Pregnant women; 15-49 years; living in Shurugwi district Zimbabwe; infants aged 1-18 months (49% female infants in larger SHINE trial, 44% female infants in microbiome sub-study)
Recruitment	Recruitment procedures have been described in detail in the study protocol (https://osf.io/mgp3t/). Briefly, village health workers from the study area sensitized potential participants to the trial, provided pregnancy tests and eligible individuals were consented. For the microbiome sub-study, sample selection was conducted to enhance longitudinal profiling of the mother and infants gut microbiota. Of the mother-infant pairs within the EED sub-study, those with least one maternal fecal specimen (of 2 possible) and at least 2 infant fecal specimens (of 5 possible) were included in the gut microbiome analyses. This selection criteria, chosen to include the most complete sample collection during follow-up, in order to strengthen our inferences about development of the gut microbiome over time, may introduce selection bias. Baseline characteristics of the microbiome sub-cohort were largely similar to those of the larger trial, suggesting that the microbiome sub-study cohort studied was largely representative of the larger SHINE trial.
Ethics oversight	All SHINE mothers provided written informed consent. The Medical Research Council of Zimbabwe (MRCZ/A/1675), Johns Hopkins Bloomberg School of Public Health (JHU IRB # 4205.), and the University of British Columbia Ethics Committee (H15-03074) approved the study protocol, including the microbiome analyses. The SHINE trial is registered at ClinicalTrials.gov (NCT01824940).

Note that full information on the approval of the study protocol must also be provided in the manuscript.

Field-specific reporting

Please select the one below that is the best fit for your research. If you are not sure, read the appropriate sections before making your selection.

☒ Life sciences ☐ Behavioural & social sciences ☐ Ecological, evolutionary & environmental sciences

For a reference copy of the document with all sections, see nature.com/documents/nr-reporting-summary-flat.pdf

Life sciences study design

All studies must disclose on these points even when the disclosure is negative.

Sample size	Sample sizes calculations have previously been published for the larger SHINE trial within which this study is based (https://osf.io/mgp3t/). This microbiome study uses a convenience sample set based on available samples and therefore no sample size calculation was performed. Sample selection is described in the methods.
Data exclusions	No data were excluded. Exclusion criteria for the larger SHINE trial have been previously described in the trial protocol (https://osf.io/mgp3t/)
Replication	A set of stool specimens (4 participants, at 3 ages) were subject to replicate DNA extraction, library preparation and sequencing in triplicate, to estimate the magnitude of technical variability among samples. Principal coordinates analysis (PCoA) of Bray-Curtis distances and phylum-level relative abundances revealed little variation between replicates. Raw sequencing data from all samples has been deposited on a publicly available repository and is available for replication.
Randomization	Cluster-randomization to the four trial arms was performed for the larger SHINE trial as previously detailed in the trial protocol (https://osf.io/mgp3t/)
Blinding	Due to the nature of the interventions (building pit latrines, hand-washing stations etc.), the trial was unblinded.

Reporting for specific materials, systems and methods

We require information from authors about some types of materials, experimental systems and methods used in many studies. Here, indicate whether each material, system or method listed is relevant to your study. If you are not sure if a list item applies to your research, read the appropriate section before selecting a response.

Materials & experimental systems

n/a	Involved in the study
☒	☐ Antibodies
☒	☐ Eukaryotic cell lines
☒	☐ Palaeontology and archaeology
☒	☐ Animals and other organisms
☐	☒ Clinical data
☒	☐ Dual use research of concern

Methods

n/a	Involved in the study
☐	☒ ChIP-seq
☐	☒ Flow cytometry
☐	☒ MRI-based neuroimaging

Clinical data

Policy information about clinical studies

All manuscripts should comply with the ICMJEguidelines for publication of clinical research and a completedCONSORT checklist must be included with all submissions.

Clinical trial registration	NCT01824940
Study protocol	https://osf.io/w93hy
Data collection	Recruitment was conducted in Shurugwi district Zimbabwe between Nov 22, 2012 and March 27, 2015. All follow-up data collection was completed by July 31, 2017. Briefly, village health workers from the study area sensitized potential participants to the trial, provided pregnancy tests and eligible individuals were consented. Data were collected in the home by study nurses.
Outcomes	Primary outcomes of the main SHINE trial (infant length-for-age Z score and haemoglobin concentrations at 18 months of age among children born to mothers who were HIV negative during pregnancy) have been previously outlined in the study protocol (https://osf.io/mgp3t/). Length-for-age Z-scores were assessed by comparing length and age of children against standardized World Health Organization growth standards. Haemoglobin concentrations were assessed using the Hemocue hemoglobinometer Outcomes of the microbiome sub-study were alpha diversity, gene richness, species/pathway relative abundance, microbiome-for-age Z-score/metagenome-for-age Z-score and XGBoost predictive strength (R2 and mean absolute error).

ChIP-seq

Data deposition

☐ Confirm that both raw and final processed data have been deposited in a public database such as GEO.

☐ Confirm that you have deposited or provided access to graph files (e.g. BED files) for the called peaks.

Data access links<br>*May remain private before publication.*	*For "Initial submission" or "Revised version" documents, provide reviewer access links. For your "Final submission" document, provide a link to the deposited data.*
Files in database submission	*Provide a list of all files available in the database submission.*
Genome browser session<br>(e.g. UCSC)	*Provide a link to an anonymized genome browser session for "Initial submission" and "Revised version" documents only, to enable peer review. Write "no longer applicable" for "Final submission" documents.*

Methodology

Replicates	*Describe the experimental replicates, specifying number, type and replicate agreement.*
Sequencing depth	*Describe the sequencing depth for each experiment, providing the total number of reads, uniquely mapped reads, length of reads and whether they were paired- or single-end.*
Antibodies	*Describe the antibodies used for the ChIP-seq experiments; as applicable, provide supplier name, catalog number, clone name, and lot number.*
Peak calling parameters	*Specify the command line program and parameters used for read mapping and peak calling, including the ChIP, control and index files used.*
Data quality	*Describe the methods used to ensure data quality in full detail, including how many peaks are at FDR 5% and above 5-fold enrichment.*
Software	*Describe the software used to collect and analyze the ChIP-seq data. For custom code that has been deposited into a community repository, provide accession details.*

Flow Cytometry

Plots

Confirm that:

- [ ] The axis labels state the marker and fluorochrome used (e.g. CD4-FITC).
- [ ] The axis scales are clearly visible. Include numbers along axes only for bottom left plot of group (a 'group' is an analysis of identical markers).
- [ ] All plots are contour plots with outliers or pseudocolor plots.
- [ ] A numerical value for number of cells or percentage (with statistics) is provided.

Methodology

Sample preparation	*Describe the sample preparation, detailing the biological source of the cells and any tissue processing steps used.*
Instrument	*Identify the instrument used for data collection, specifying make and model number.*
Software	*Describe the software used to collect and analyze the flow cytometry data. For custom code that has been deposited into a community repository, provide accession details.*
Cell population abundance	*Describe the abundance of the relevant cell populations within post-sort fractions, providing details on the purity of the samples and how it was determined.*
Gating strategy	*Describe the gating strategy used for all relevant experiments, specifying the preliminary FSC/SSC gates of the starting cell population, indicating where boundaries between "positive" and "negative" staining cell populations are defined.*

- [ ] Tick this box to confirm that a figure exemplifying the gating strategy is provided in the Supplementary Information.

Magnetic resonance imaging

Experimental design

Design type	*Indicate task or resting state; event-related or block design.*
Design specifications	*Specify the number of blocks, trials or experimental units per session and/or subject, and specify the length of each trial or block (if trials are blocked) and interval between trials.*
Behavioral performance measures	*State number and/or type of variables recorded (e.g. correct button press, response time) and what statistics were used to establish that the subjects were performing the task as expected (e.g. mean, range, and/or standard deviation across subjects).*

Acquisition

Imaging type(s)	*Specify: functional, structural, diffusion, perfusion.*
Field strength	*Specify in Tesla*
Sequence & imaging parameters	*Specify the pulse sequence type (gradient echo, spin echo, etc.), imaging type (EPI, spiral, etc.), field of view, matrix size, slice thickness, orientation and TE/TR/flip angle.*
Area of acquisition	*State whether a whole brain scan was used OR define the area of acquisition, describing how the region was determined.*

Diffusion MRI ☐ Used ☐ Not used

Preprocessing

Preprocessing software	*Provide detail on software version and revision number and on specific parameters (model/functions, brain extraction, segmentation, smoothing kernel size, etc.).*
Normalization	*If data were normalized/standardized, describe the approach(es): specify linear or non-linear and define image types used for transformation OR indicate that data were not normalized and explain rationale for lack of normalization.*
Normalization template	*Describe the template used for normalization/transformation, specifying subject space or group standardized space (e.g. original Talairach, MNI305, ICBM152) OR indicate that the data were not normalized.*
Noise and artifact removal	*Describe your procedure(s) for artifact and structured noise removal, specifying motion parameters, tissue signals and physiological signals (heart rate, respiration).*

Volume censoring | *Define your software and/or method and criteria for volume censoring, and state the extent of such censoring.*

Statistical modeling & inference

Model type and settings | *Specify type (mass univariate, multivariate, RSA, predictive, etc.) and describe essential details of the model at the first and second levels (e.g. fixed, random or mixed effects; drift or auto-correlation).*

Effect(s) tested | *Define precise effect in terms of the task or stimulus conditions instead of psychological concepts and indicate whether ANOVA or factorial designs were used.*

Specify type of analysis: [ ] Whole brain [ ] ROI-based [ ] Both

Statistic type for inference
(See Eklund et al. 2016) | *Specify voxel-wise or cluster-wise and report all relevant parameters for cluster-wise methods.*

Correction | *Describe the type of correction and how it is obtained for multiple comparisons (e.g. FWE, FDR, permutation or Monte Carlo).*

Models & analysis

n/a	Involved in the study
[ ]	[ ] Functional and/or effective connectivity
[ ]	[ ] Graph analysis
[ ]	[ ] Multivariate modeling or predictive analysis

Functional and/or effective connectivity | *Report the measures of dependence used and the model details (e.g. Pearson correlation, partial correlation, mutual information).*

Graph analysis | *Report the dependent variable and connectivity measure, specifying weighted graph or binarized graph, subject- or group-level, and the global and/or node summaries used (e.g. clustering coefficient, efficiency, etc.).*

Multivariate modeling and predictive analysis | *Specify independent variables, features extraction and dimension reduction, model, training and evaluation metrics.*
